# Prevalence and correlates of tobacco use amongst junior collegiates in twin cities of western Nepal: A cross-sectional, questionnaire-based survey

**DOI:** 10.1186/1471-2458-8-97

**Published:** 2008-03-26

**Authors:** Chandrashekhar T Sreeramareddy, PV Kishore, Jagadish Paudel, Ritesh G Menezes

**Affiliations:** 1 Department of Community Medicine, Manipal Teaching Hospital, Manipal College of Medical Sciences, Pokhara, Nepal; 2 Department of Internal Medicine, Manipal Teaching Hospital, Manipal College of Medical Sciences, Pokhara, Nepal; 3 Department of Sociology, Prithvi Narayan Campus, Pokhara, Nepal; 4 Department of Forensic Medicine and Toxicology, Kasturba Medical College, Mangalore, India

## Abstract

**Background:**

College students are vulnerable to tobacco addiction. Tobacco industries often target college students for marketing. Studies about prevalence of tobacco use and its correlates among college students in Nepal are lacking.

**Methods:**

A cross-sectional survey was carried out in two cities of western Nepal during January-March, 2007. A pre-tested, anonymous, self-administered questionnaire (in Nepali) adapted from Global Youth Tobacco Survey (GYTS) and a World Bank study was administered to a representative sample of 1600 students selected from 13 junior colleges by two-stage stratified random sampling.

**Results:**

Overall prevalence of 'ever users' of tobacco products was 13.9%. Prevalence among boys and girls was 20.5% and 2.9% respectively. Prevalence of 'current users' was 10.2% (cigarette smoking: 9.4%, smokeless products: 6.5%, and both forms: 5.7%). Median age at initiation of cigarette smoking and chewable tobacco was 16 and 15 years respectively. Among the current cigarette smokers, 58.7% (88/150) were smoking at least one cigarette per day. Most (67.8%) 'Current users' purchased tobacco products by themselves from stores or got them from friends. Most of them (66.7%) smoked in tea stalls or restaurants followed by other public places (13.2%). The average daily expenditure was 20 Nepalese rupees (~0.3 USD) and most (59%) students reported of having adequate money to buy tobacco products. Majority (82%) of the students were exposed to tobacco advertisements through magazines/newspapers, and advertising hoardings during a period of 30 days prior to survey. The correlates of tobacco use were: age, gender, household asset score and knowledge about health risks, family members, teachers and friends using tobacco products, and purchasing tobacco products for family members.

**Conclusion:**

School/college-based interventions like counseling to promote cessation among current users and tobacco education to prevent initiation are necessary. Enforcement of legislations to decrease availability, accessibility and affordability of tobacco products and policies to change social norms of tobacco use among parents and teachers are necessary to curb the tobacco use among college students.

## Background

Tobacco use is one of the leading preventable causes of premature death, disease and disability around the world [1]. An estimated 4.9 million deaths occurring annually can be attributed to tobacco use. This figure is expected to rise to about 10 million by the year 2020, if the current epidemic continues and more than 70% of these deaths are expected to occur in developing countries [2]. In Nepal, chronic non-communicable diseases account for 42% of all the deaths [3]. This rate may be due to a high prevalence of current daily tobacco users among men and women aged 18 years and above which was 48.5% and 24% respectively [4]. In the mountain region, the reported prevalence of females smoking was 71.6% [5]. Various studies have reported prevalence ranging from 20% to 72% in different populations of Nepal [5-8]. A study from eastern Terai reported a prevalence of 12.9% and 14.1% respectively for cigarette smoking and smokeless tobacco use among women [8]. The Global Tobacco Surveillance System (GTSS) collaborative group has reported that tobacco use (both cigarette and smokeless tobacco) among those aged 13–15 years is high and has emphasized that interventions are necessary for the prevention of initiation and to promote cessation of tobacco among current users [9]. The GYTS, Nepal (2001) has reported a prevalence of 16.3% for any form of tobacco use among the high schools students of central region [10].

Adolescents and young adults of colleges are often targeted by the tobacco industry for marketing. Moreover, college age is a transition period and students are vulnerable to tobacco addiction. Like high schools even colleges can be targeted for tobacco control interventions. A study from India reported that the prevalence of tobacco use is high and popular perceptions exist about tobacco products among students [11]. Studies about tobacco use and its correlates among college students in Nepal are lacking. Studies about awareness/knowledge of the risks of smoking are also lacking. Moreover, the previous studies including GYTS, Nepal [10] have not explored the factors determining tobacco use behavior among college students. A World Bank study from Indonesia identified various predisposing, enabling and reinforcing factors which determined tobacco use among the Indonesian youth in high schools [12]. These factors may be specific to culture, traditions and other characteristics of a country. Identification of such factors may be potentially useful to formulate policy interventions needed towards behavior change communication for prevention and control of tobacco use among college students. Therefore, we carried out the present study with the following objectives:

1. to estimate the prevalence of tobacco use, and

2. to assess the correlates of tobacco use.

## Methods

### Study area

Nepal is a poor developing country in the South-east Asia region of the World Health Organization (WHO). In 2002, Nepal was ranked 140^th ^among 174 countries in the Human Development Index and with a Gross Domestic Product 0.44 [13]. It has a total land area of 147,000 square kilometers. According to the 2001 census, the total population of Nepal was 23.15 million (male: 11.56 million and female: 11.58 million) and the sex-ratio was 998 males for 1000 females. As a consequence of a high growth rate, majority of population of the country is fairly young. About 39.3% of the total population is in the age group 0–14 years and only 6.5% are above 60 years of age. Eighty-one per cent of the economically active population is employed in the agricultural sector. Nepal is a proclaimed Hindu Kingdom. Eighty percent of the population is Hindus, followed by Buddhists (10.7%) and Muslims (4.2%). Only 48.1% of males above 14 years of age have minimum high school level education while for females the percentage was 27.2. Kaski district is one of the 14 districts in western development region of Nepal. The district has a land area of 2017 square kilometers and a population of 380527. Kaski district has 43 village development committees, Pokhara sub metropolitan city and Leknath municipality whose populations are 156312 and 41369 respectively [14].

### Conceptual frame work of the study

We followed the same conceptual frame work as the World Bank study on tobacco economics carried out in Indonesia (2003) [12]. This study was based on Green's (1991) PRECEDE (Predisposing, reinforcing and enabling factors in communication and educational diagnostic evaluation) model [15]. According to the PRECEDE model, three important groups of factors play an important role in changing the behavior and the environment. These are:

1) Predisposing factors,

2) Enabling factors, and

3) Reinforcing factors.

Based on these factors educational and organizational strategies can be implemented for behavior and environment change [15].

#### Study design

A cross-sectional, anonymous self-administered questionnaire survey was carried out.

### Questionnaire

The English versions of the questionnaires used for the Global Youth Tobacco Survey, 2001 [16] and World Bank study on tobacco economics, Indonesia, 2003 [12] were reviewed. The authors were contacted and permission was obtained to use the questionnaire for this survey. The questionnaire was adapted and modified from those mentioned above to suit the cultural sensitivity of Nepal. The questionnaire thus prepared in English was translated into local vernacular language, Nepali by JP (Jagadish Paudel). The Nepalese version of questionnaire was pre-tested among 50 students of a publicly-funded junior college. The necessary modifications were made after pretesting. The final version of the questionnaire was back-translated into English by two third year undergraduate Nepalese medical students. The questionnaire contained items on demographic data, age at initiation, frequency and type of tobacco products used. The questionnaire also contained questions on knowledge about the health risks of tobacco use, beliefs and attitudes towards tobacco use, tobacco use habits among friends, family members and teachers, exposure to tobacco advertisements and efforts towards cessation of tobacco use. The questions about tobacco-related school curriculum and exposure to environmental tobacco smoke from GYTS questionnaire were not included.

### Definitions of the variables

#### Ever users

Ever smoker or chewer was defined as one who had not smoked/chewed tobacco in the past 30 days preceding the survey but had tried in the past (even once or twice).

#### Current tobacco user

Current smoker or chewer was defined as those who had smoked/chewed tobacco product on one or more days in the preceding month of the survey.

#### Household asset score and categories

We used household asset score as a proxy to the economic status of the students. Eight items: radio, bicycle, television, fridge, motor-bike, washing machine, computer and car present at their homes were indicated in the demography section of the questionnaire. Each item was given a score ranging from 5 to 40 with a maximum total score of 180 if all the seven items were present (For example, radio was given least score of 5 and car was given the highest score of 40). For each student the total score was calculated and divided into one of the three categories i.e. low (0–60) middle (61–120) and high (121–180).

#### Knowledge score

Ten questions on knowledge about the harmful effects of smoking were included in the questionnaire. For each question correct response was given a score of one and incorrect or 'don't know' was scored as zero. The sum giving a minimum score of zero and a maximum of 10 was used as a continuous variable.

### Sample size determination and sampling method

The GYTS, Nepal (2001) reported that overall 16.3% of the students belonging to 8^th ^– 10^th ^grade had ever used tobacco product in any form [10]. Among the junior college students (11^th ^and 12^th ^grades) we expected a minimum prevalence of tobacco use to be 20%. The required minimum sample size to estimate the prevalence was calculated for 95% confidence limits and an allowable error of 10% was 1600.

A two stage stratified random sampling method was used to select a representative sample of students. There were seven junior colleges in Leknath municipality and 30 in Pokhara Sub Metropolitan City. A list of all the junior colleges and their enrollment sizes was obtained from the district secondary education board. At the first stage, five publicly-funded colleges and eight private colleges were randomly selected by stratification according to type of college (private/public) from both the cities. The college enrollment size varied from 25 in private colleges to approximately 6,000 in publicly-funded colleges. Publicly-funded colleges had the largest enrollment size. In the second stage, in colleges with an enrollment size of 100 or less, all the students were included. Two or more classes were randomly selected by lottery method from those colleges with an enrollment size of more than 100. The largest of all the colleges was Prithvi Narayan Campus affiliated to Tribhuvan University. The college had about approximately 6000 students in different streams of study. Therefore, five classes belonging to different streams of study were selected for survey.

### Data Collection

A written permission was obtained from the office of the district secondary education board. The research ethics committee of the Manipal College of Medical Sciences approved this study. The survey was carried out between January and April, 2007. The researchers (CTS and JP) were involved in administering the survey instrument (questionnaire) to the students. The principals/institutional chiefs of the selected college were personally contacted by the researchers. The objective and nature of the study was explained and a verbal consent was sought to carry out the survey in the college. All the students present at the time of our visit were included for the survey. The students of the selected class were assembled in their class room. The purpose of the survey was explained and assurance about the confidentiality of the information provided was given to the students. After such briefing, they were invited to participate in the survey. Informed consent of the students was sought and the students were informed that they were free to opt not to participate in the survey. Absence of the school personnel/teachers in the classrooms was ensured to encourage the students respond without reporting bias. The printed copies of the anonymous, self-administered questionnaires in Nepali were distributed to the students. The completed questionnaires were collected by the researchers.

### Data Analysis

Data were coded and entered into Microsoft excel package and extracted into SPSS (Statistical Package for Social Sciences) version 10 for windows. Rates, percentages and descriptive statistics were calculated. The 'ever use' of tobacco products in any form was used as dependant variable. Demographic characteristics, predisposing, enabling and reinforcing factors were treated as independent variables. Multivariate logistic regression analyses were carried out by 'enter' method. All the groups of variables were entered into the regression model. Odds ratios (OR), 95% confidence intervals (95% CI) and p-values were calculated for each independent variable.

## Results

All the colleges contacted responded to the survey giving a college response rate of 100%. A total of 1662 students, were selected and invited to participate in the survey. Out of these 1596 students completed the questionnaires giving a response rate of 96%. In six questionnaires the responses were incomplete and therefore excluded from analysis. The final analysis was carried out for 1590 questionnaires.

### Demographic characteristics

The mean age of the students was 17.8 years (SD, 1.7). The median age was 17 years (minimum 14 years and maximum 32 years). The male to female ratio was 1.6:1. Out of 1590 students, 996 (62.6%) were males and 594 (37.4%) were females. A majority (1383/1590, 87%) of the students were Hindus, followed by Buddhists (157/1590, 9.9%).

### Prevalence of tobacco use

**Figure 1:**
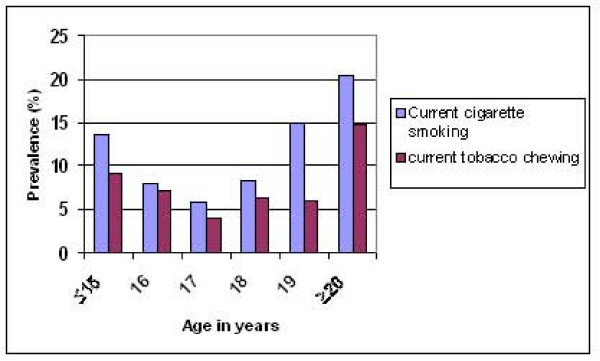
Prevalence of current tobacco use according to age.

The prevalence of tobacco use according to gender is shown in Table 1. Overall prevalence of 'ever users' of tobacco products was 13.9%. Prevalence among boys and girls was 20.5% and 2.9% respectively. Prevalence of 'current use' of tobacco products was 10.2% (cigarette smoking: 9.4%, smokeless products: 6.5%, both forms: 5.7%). Majority of 'current users' of any tobacco product were 17 years or older. The prevalence of 'current users' increased after 17 years, the highest being among students who were 20 years or older (Figure 1). The differences in prevalence according to gender (p < 0.001) and age (p < 0.001) were statistically significant.

**Table 1:** Prevalence of tobacco use (%)

	Male* (N = 996)	Female* (N = 594)	Overall* (N = 1590)
Ever used any tobacco product	20.5 (17.9,22.9)	2.9 (1.5,4.2)	13.9 (12.2,15.6)
Currently smoking cigarettes	14 (11.8,16.1)	1.9 (0.8,2.9)	9.4 (8,10.9)
Currently using chewable tobacco	9.3 (7.5,11.1)	1.9 (0.8,2.9)	6.5 (5.3,7.8)
Currently using both forms of tobacco product	8.2 (6.5,9.9)	1.5 (0.5,2.5)	5.7 (4.6,6.9)

* 95% confidence intervals

### Age at initiation of tobacco use

**Figure 2:**
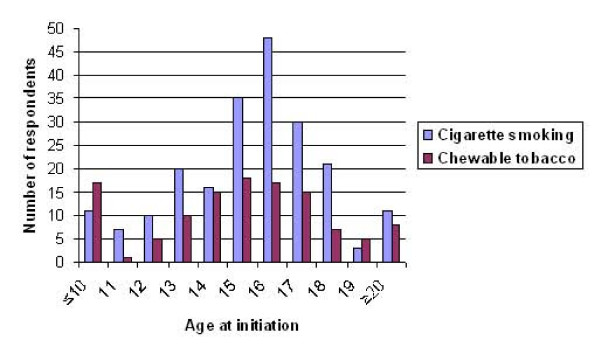
Age at initiation of tobacco use.

Figure 2 shows age at initiation of tobacco use. Majority (89.6%) of ever smokers had initiated smoking between the ages 12 and 18 years with a median age of 16 years. Among 'ever users' of chewable tobacco, 73.7% had initiated between the ages 12 and 18 years with a median age of 15 years. Among ever smokers, 30.2% had initiated before 15 years of age and 36.4% of 'ever users' of chewable tobacco had initiated before 15 years of age.

### Smoking pattern and access to tobacco products

Among the current cigarette smokers, 58.7% (88/150) were smoking at least one cigarette per day, 27.3% (41/150) smoked once or twice a week and 14% (21/150) smoked once or twice in a month. The average number of cigarettes smoked in a day was 3.5 (median 2, mode 1). About 62% of the students smoked one or two cigarettes per day. Most of them (66.7%) smoked in tea stalls or restaurants followed by other public places (13.2%). The others smoked at home (7.4%) or a 'secret place' (15.2%). Most (67.8%) of them had purchased tobacco products from a shop or street vendor by themselves while 11.8% of the students purchased through a friend and 10.3% borrowed from friends. Six students also reported that they obtained tobacco products from a family member. Among the 104 current tobacco chewers, 34 (32.7%) were using *'gutka' *(mixture of tobacco and molasses), 27 (25%) *'pan masala' *(tobacco with aromatic spices) 24 (23.1%) *'surti' *(dried tobacco leaves for chewing), 12 (11.5%) '*khaini' *(tobacco with slaked lime and aromatic spices) and the remaining were using combinations of different types of chewable tobacco.

About 146 'current users' of tobacco products had responded for the average amount of money spent per day for buying tobacco products. They spent an average amount of 20 Nepalese rupees (NRS) [0.3 USD] per day (SD 23.6 NRS) (1 USD≈66 NRS). The amount spent varied from 3 NRS [0.04 USD] to 130 NRS [1.96 USD] with a median of 10 NRS [0.15 USD] (quartiles, 5 and 20). Out of 200 students who responded for the question "Do you have sufficient amount to buy tobacco products? 118 (59%) had replied 'yes'.

### Media and advertising

About 82% of the students had seen tobacco-related advertisements during the preceding 30 days of the survey. Magazines (59.7%), newspapers (56.9%), television (48.7%), radio (41.9%) and advertisement hoardings (39.2%) were the media from which students were exposed to tobacco advertisements most often. Seven percent of the students had something (T-shirt, pen, bag etc) which had a cigarette brand logo on it. About 963 (60.6%) students believed that cigarette advertisement or promotion can influence smoking use among students.

### Cessation

The experiences of the current tobacco users of about cessation are shown in Table 2. A larger proportion of the students who were either currently smoking (66.6%) or using chewable tobacco (62.5%) thought that they can quit, whereas a lesser proportion (63.3% and 57.7% respectively) had actually tried to quit. However, 55.3% and 49.1% of the students who were currently smoking and chewing tobacco respectively had ever sought help to quit tobacco products. In most instances they sought advice from a friend or a girlfriend.

**Table 2:** Experiences of the current tobacco users about cessation

**Experience**	**Smokers (N = 150)**	**Chewable tobacco (N = 104)**
	
	**Number of students (%)**	**Number of students (%)**
Had tried to quit	95 (63.3)	60 (57.7)
Thinks that he/she can quit	100 (66.6)	65 (62.5)
Sought help to quit	83 (55.3)	51(49.1)

### Correlates of tobacco use

The results of the multivariate logistic regression analysis are shown in Table 3. Among the demographic factors, being of older age and being a male were associated with tobacco use. Household asset score was used as a proxy for economic status. Students who had high asset score were more likely to be ever users of tobacco. Among predisposing factors knowledge about harm of tobacco use was protective for tobacco use. Among the enabling and reinforcing factors, tobacco use among the family members, friends and teachers were associated with tobacco use among the students. Students who had ever purchased tobacco products for their family members were more likely to have used any tobacco product.

**Table 3:** Multivariate analysis of determinants of tobacco use

**Variable**	**Number**	**Adjusted OR**	**95% C I**	**p-value**
**Age (Mean, SD)**	178, 1.7	**1.13**	**1.04, 1.22**	**0.004**
**Gender**				
Female	587	1		
Male	974	**7.03**	**4.03, 12.28**	**<0.001**
**Religion**				
Others	206	1		
Hindu	1355	1.42	0.86, 2.35	0.162
**Currently residence**				
Hostel/rented room	679	1		
With parents	882	0.98	0.71, 1.36	0.93
**Household asset score**				
Low	1065	1		
Medium	394	1.71	0.95, 3.07	0.72
High	102	**1.52**	**1.06, 2.12**	**0.03**
**Are Tobacco products available at any place and anytime?**				
No/don't know	679	1		
Yes	882	0.86	0.62, 1.21	0.38
**Is the use of tobacco products banned in your college premises?**				
No/don't know	624	1		
Yes	937	1.1	0.79, 1.53	0.59
**Do you think that your teachers smoke or chew tobacco?**				
No/don't know	499	1		
Yes	1109	**1.93**	**1.38, 2.71**	**<0.001**
**Do your close friends smoke or chew tobacco?**				
No/don't know	871	1		
Yes	690	**1.69**	**1.20, 2.38**	**0.003**
**Number family members who use tobacco**		**1.40**	**1.12, 1.76**	**0.003**
**Have you ever or currently buy tobacco products for your members?**				
No	1109	1		
Yes	452	**1.78**	**1.28, 2.48**	**0.001**
**Knowledge score (harms of tobacco use)**		**0.75**	**0.68, 0.83**	**<0.001**
**In your opinion do tobacco-related advertisements influence tobacco consumption?**				
No/don't know	610	1		
Yes	951	0.81	0.58, 1.13	0.214

Only 1561 cases were included in the final logistic regression due to missing data for some variables.

## Discussion

The dual purposes our study were to estimate the prevalence of tobacco use and assess the correlates. The sample size was determined using data from GYTS, Nepal and obtained a representative sample from the twin cities. We used a standardized survey instrument from GYTS [16] which made our data comparable to GYTS reports. However, our study was carried out among the college students instead of high school students in GYTS. In our study, the median age was 17 years which was higher than that of GYTS surveys. Despite this, the prevalence of ever tobacco use was slightly lesser than that reported by the GYTS, Nepal [10]. GYTS was carried out in central region which included both urban and rural areas but our study was carried out in urban area only. However, the prevalence of our study is lesser than that reported from Kerala, India [17] and USA where nearly one-third of the students were currently using tobacco products [18]. It is important to note the prevalence was low compared to western countries which have strict legislation, heavy taxes and massive information campaigns. Such difference was found in GYTS survey also [9]. Prevalence lower than that of western countries despite the lack of tobacco control measures may be explained by differences in socio-cultural milieu between these populations.

Globally, GYTS reports have shown that the difference among current smokers between boys and girls is smaller than that between men and women [9,19]. However, there was a yawning gap in prevalence between boys and girls in our study. A community-based survey from Eastern Nepal reported a similar rate of prevalence in the age group 14–25 years. However, the study reported that the prevalence of smoking increased with age [8]. Similar gender differentials and increasing trends of tobacco use with age was reported by WHO [20].

The study from Kerala, India has reported that the age at initiation appears to be declining [17]. The results of our study are similar to those reported from Kerala, India [17] and Indonesia [12]. The age at initiation for chewable products was lesser than that for smoking. This may be due to the growing popularity of the smokeless products accessible and available in small attractive sachets [21]. A good proportion (5.7%) of students was currently using both forms of tobacco. It appears that smoking is often preceded by the use of chewable tobacco. The broad use of tobacco products among a substantial portion of this 16–20 years age group could be an indication of a future increase in overall adult tobacco use. Less than half the students either tried to quit tobacco use or sought professional help to quit tobacco use. Therefore, counseling and quit-line programmes need to be started at the colleges for the benefit of the current users.

Most students (78.7%) thought that street vendors, tea stalls and shops are selling tobacco products and 63.3% of the students responded that tobacco products are easily available. It has been suggested that banning the sales of tobacco products may be a deterrent to tobacco use among youth. However, there is no such legislation existing in Nepal [22]. Most students purchased tobacco products by themselves from street vendors or shops and were not denied by the virtue of their age. Average number of cigarettes smoked per day was about three. About three-fifths of the students smoked one or two cigarettes per day. One of the measures to control tobacco use is the ban of single stick sale. Such legislation also does not exist in Nepal [22]. The common place of smoking was tea stall or restaurants and public places. Legislations like restricted sales of tobacco products and ban of smoking in public places might be helpful in curbing the tobacco use among these young adults. A majority of the students had seen pro-tobacco advertisements during the preceding 30 days from a wide range of media. However, this was not significantly different between ever tobacco users and never users. In Nepal there is no existing legislation about sampling and sponsoring by the tobacco industry [22]. There should be a complete ban of tobacco advertisements as partial ban has shown to be ineffective [23]. The WHO Framework Convention on Tobacco Control (FCTC) requires a comprehensive ban on advertising and promotion which may be ratified by the public health authorities in Nepal [24]. Measures like banning tobacco use in movies and television are thought to be sound public health policies as youth are influenced by movies and television [25].

The independent factors determining tobacco use among these college students were age, gender and household assets. Students from higher income group may be getting higher pocket money and therefore could afford to buy tobacco products. Among the other factors knowledge score was protective for tobacco use. Those students who had better knowledge of health risks of tobacco use were less likely to have 'ever used' any tobacco products. Similar observations were made in studies from Indonesia [12], and Argentina [26]. Therefore, it may be beneficial to introduce lessons on health risks of tobacco at schools and colleges.

Among the reinforcing factors the students who reported that one or more family members smoking or chewing tobacco were more likely to be ever users of tobacco. We investigated tobacco use among parents and siblings. As the number of family members using tobacco increased by a unit, the risk of tobacco used increased 1.5 times. Similarly having purchased tobacco products for a family member was also associated with tobacco use. The students who had friends who used tobacco products and who reported that their teachers use tobacco products were also likely to use tobacco products. Similar results have been reported by studies from Indonesia [12], Argentina [26] and Kerala, India [17]. Even school children have opined that parents [27] and peers have strong influence on tobacco use amongst youth [28]. However, there is a possibility that tobacco users may have reported about users in their environment due to selective perception.

Nearly 40% reported that at least one family member uses tobacco. Despite schools and colleges of Nepal being tobacco-free, the teachers' tobacco use behavior may have influenced students. Tobacco use among the family members, in particular parents and siblings may also have an influence. Similarly in the college environment peer pressure also appears to play a role for adopting tobacco use as a way of life. Tobacco use among adults, lack of ban on smoking in public places, exposure to tobacco use at home may have created an opinion that tobacco use is a 'social norm'. Behavior change communication may not be successful unless adults change their behavior. Despite good knowledge about health risks of tobacco use among all the students, their perception of individual risk may be poor. Educational efforts should focus on dispelling the misconceptions created in the young minds by the media.

## Limitations

Our study had some limitations. Our survey was cross-sectional, and smoking status was by self-report. Therefore, some students may have under reported their tobacco use. Moreover the existing taboo about tobacco use, some female students in particular might also be underreported. Some students who were using tobacco might have been absent from the college on the day of survey. We did not have an opportunity to interview them. The study was carried out in colleges of two urban locations only. So our results cannot be extrapolated to the rest of Nepal.

## Conclusion

Tobacco use in any form (smoking or smokeless) is prevalent among the college students. Cigarette smoking was the most popular form of tobacco use. Older male college students were more likely to use tobacco. Knowledge of health risk, household asset score, peer influences and social norms like tobacco use among teachers and family members, buying tobacco products for a family member were associated with tobacco use. Targeted school/college-based intervention strategies by counseling and education are necessary. Enforcement of regulations on sale and advertisements of tobacco products may also be useful. Legislations on use of tobacco products need to be enforced to decrease availability, accessibility and affordability of tobacco products. Policies to bring about changes in acceptability of tobacco use (social norms) among parents, teachers may also help to curb the tobacco use among college students.

## Competing interests

The author(s) declare that they have no competing interests.

## Authors' contributions

CTScontributed to the design and protocol of the study, participated in the data collection, was the primary researcher and drafted the manuscript for publication. PVKhelped with the design of the study, data analysis, development of questionnaire and assisted in preparation of first draft. JPDesigned and conducted the data analysis, assisted in manuscript preparation and criticized the earlier drafts of the manuscript. RGMconceived the study, set up the design, development of questionnaire and criticized the earlier drafts of the manuscript. All authors read and approved the final manuscript for submission for publication.

## Pre-publication history

The pre-publication history for this paper can be accessed here:



## References

[B1] Ezzati M, Lopez AD, Rodgers A, Vander Hoorn S, Murray CJL, the Comparative Risk Assessment Collaborating Group (2002). Selected major risk factors and global and regional burden of disease. Lancet.

[B2] Peto R, Lopez AD, Boreham J, Thun M, Heath C (1994). Mortality from smoking in developed countries 1950–2000: indirect estimation from National Vital Statistics.

[B3] World Health Organization (2002). The impact of chronic diseases in Nepal. WHO.

[B4] WHO South-East Asia Region (2001). WHO World Health Survey. WHO Global InfoBase Version: 1292beta.

[B5] Pandey MR, Neupane RP, Gautam A (1988). Epidemiological study of tobacco smoking behaviour among adults in a rural community of the hill region of Nepal with special reference to attitude and beliefs. Int J Epidemiol.

[B6] Pandey MR, Venkatramaiah SR, Neupane RP, Gautam A (1987). Epidemiological study of tobacco smoking behaviour among young people in a rural community of the hill region of Nepal with special reference to attitude and beliefs. Community Med.

[B7] Jha N, Upadhyay MP, Lakhey S, Yadav BK, Baral DD, Ghartichhetri PS (1999). Smoking and smokers in Sunsari, Nepal. J Nep Med Assoc.

[B8] Niraula SR (2004). Tobacco use among women in Dharan, eastern Nepal. J Health Popul Nutr.

[B9] Warren CW, Jones NR, Eriksen MP, Asma S, Global Tobacco Surveillance System (GTSS) collaborative group (2006). Patterns of global tobacco use in young people and implications for future chronic disease burden in adults. Lancet.

[B10] Pandey MR, Pathak RP (2002). Challenges of tobacco use behavior in central development region of Nepal: Global Youth Tobacco Survey Collaborative Group. Nepal GYTS Fact Sheet.

[B11] Nichter M, Nichter M, Van Sickle D (2004). Popular perceptions of tobacco products and patterns of use among male college students in India. Soc Sci Med.

[B12] Martini S, Sulistyowati M (2003). The Determinants of Smoking Behavior among Teenagers in East Java Province, Indonesia. Health, Nutrition and Population (HNP) Discussion Paper Economics Of Tobacco Control Paper No 32 World Bank.

[B13] United Nations (UN) (2002). Human Development Report.

[B14] Central Bureau of Statistics, National Planning Commission Secretariat (2001). Population Census 2001.

[B15] Green L, Kreuter M (1991). An Educational and Environmental Approach. Health Promotion Planning.

[B16] Center for Disease Control and Prevention Global Youth Tobacco Survey. http://www.cdc.gov/tobacco/global/GYTS/intro.htm.

[B17] Pradeepkumar AS, Mohan S, Gopalakrishnan P, Sarma PS, Thankappan KR, Nichter M (2005). Tobacco use in Kerala: findings from three recent studies. Natl Med J India.

[B18] Rigotti NA, Lee JE, Wechsler H (2000). US college students' use of tobacco products: results of a national survey. JAMA.

[B19] WHO (1997). Tobacco or health: a global status report.

[B20] Pande B, Karki Y, Plant K, Strong K, Bonita R (2001). A study on tobacco economics in Nepal. The SuRF Report 1 Surveillance of Risk Factors related to Noncommunicable Diseases: Current status of global data.

[B21] Shimkhada R, Peabody JW (2003). Tobacco control in India. Bull World Health Organ.

[B22] World Bank. South-east Asia region (2001). N epal smoking prevalence tobacco economy. World Bank.

[B23] Jha P, Chaloupka FJ (1999). Curbing the epidemic: Governments and the economics of tobacco control.

[B24] World Health Organization Updated status of the WHO framework convention on tobacco control. http://www.who.int/tobacco/framework/countrylist/en/index.html.

[B25] Reddy KS, Arora M (2005). Ban on tobacco use in films and television represents sound public health policy. Natl Med J India.

[B26] Morello P, Duggan A, Adger H, Anthony JC, Joffe A (2001). Tobacco use among high school students in Buenos Aires, Argentina. Am J Public Health.

[B27] Staten RR, Noland M, Rayens MK, Hahn E, Dignan M, Ridner SL (2007). Social influences on cigarette initiation among college students. Am J Health Behav.

[B28] Mishra A, Arora M, Stigler MH, Komro KA, Lytle LA, Reddy KS, Perry CL (2005). Indian youth speak about tobacco: results of focus group discussions with school students. Health Educ Behav.

